# Frequency, Severity, and Prediction of Tuberculous Meningitis Immune Reconstitution Inflammatory Syndrome

**DOI:** 10.1093/cid/cis899

**Published:** 2012-10-24

**Authors:** Suzaan Marais, Graeme Meintjes, Dominique J. Pepper, Lori E. Dodd, Charlotte Schutz, Zahiera Ismail, Katalin A. Wilkinson, Robert J. Wilkinson

**Affiliations:** 1 Clinical Infectious Diseases Research Initiative, Institute of Infectious Diseases and Molecular Medicine, University of Cape Town; 2 Infectious Diseases Unit, GF Jooste Hospital, Cape Town; 3 Department of Medicine, University of Cape Town, South Africa; 4 Department of Medicine, Imperial College London, United Kingdom; 5 Department of Medicine, University of Mississippi Medical Center, Jackson; 6 Biostatistics Research Branch, National Institute of Allergy and Infectious Diseases, National Institutes of Health, Bethesda, Maryland; 7 Division of Mycobacterial Research, MRC National Institute for Medical Research, London, United Kingdom

**Keywords:** meningitis, tuberculosis, neutrophils, pathogenesis

## Abstract

***Background.*** Tuberculosis immune reconstitution inflammatory syndrome (IRIS) is a common cause of deterioration in human immunodeficiency virus (HIV)–infected patients receiving tuberculosis treatment after starting antiretroviral therapy (ART). Potentially life-threatening neurological involvement occurs frequently and has been suggested as a reason to defer ART.

***Methods.*** We conducted a prospective study of HIV-infected, ART-naive patients with tuberculous meningitis (TBM). At presentation, patients started tuberculosis treatment and prednisone; ART was initiated 2 weeks later. Clinical and laboratory findings were compared between patients who developed TBM-IRIS (TBM-IRIS patients) and those who did not (non-TBM-IRIS patients). A logistic regression model was developed to predict TBM-IRIS.

***Results.*** Forty-seven percent (16/34) of TBM patients developed TBM-IRIS, which manifested with severe features of inflammation. At TBM diagnosis, TBM-IRIS patients had higher cerebrospinal fluid (CSF) neutrophil counts compared with non-TBM-IRIS patients (median, 50 vs 3 cells ×10^6^/L, *P* = .02). *Mycobacterium tuberculosis* was cultured from CSF of 15 TBM-IRIS patients (94%) compared with 6 non-TBM-IRIS patients (33%) at time of TBM diagnosis; relative risk of developing TBM-IRIS if CSF was *Mycobacterium tuberculosis* culture positive = 9.3 (95% confidence interval [CI], 1.4–62.2). The combination of high CSF tumor necrosis factor (TNF)-α and low interferon (IFN)-γ at TBM diagnosis predicted TBM-IRIS (area under the curve = 0.91 [95% CI, .53–.99]).

***Conclusions.*** TBM-IRIS is a frequent, severe complication of ART in HIV-associated TBM and is characterized by high CSF neutrophil counts and *Mycobacterium tuberculosis* culture positivity at TBM presentation. The combination of CSF IFN-γ and TNF-α concentrations may predict TBM-IRIS and thereby be a means to individualize patients to early or deferred ART.

## Abstract (precis)

Tuberculous meningitis (TBM) immune reconstitution inflammatory syndrome is a severe complication of antiretroviral therapy in human immunodeficiency virus–associated TBM. We found that high cerebrospinal fluid neutrophil counts and *Mycobacterium tuberculosis* culture positivity at TBM presentation characterize, and cytokine concentrations predict, this syndrome.

Paradoxical tuberculosis immune reconstitution inflammatory syndrome (IRIS) occurs in 8%–43% of human immunodeficiency virus (HIV)–infected patients receiving tuberculosis treatment after starting antiretroviral therapy (ART) [1–4]. Tuberculosis IRIS results from rapid restoration of *Mycobacterium tuberculosis*–specific immune responses, but its pathogenesis remains poorly understood [1, 5, 6].

Neurological tuberculosis IRIS occurs in a substantial proportion (12%) of tuberculosis IRIS cases and is the commonest cause of central nervous system (CNS) deterioration during the first year of ART in settings of high tuberculosis/HIV prevalence [7, 8]. Mortality is high (up to 30%) in those affected [8]. Manifestations of neurological tuberculosis IRIS include meningitis [7–11], intracranial tuberculomata [7, 8, 12–14], brain abscesses [12, 15], radiculomyelitis [7, 8, 11], and spinal epidural abscesses [7]. There are no prospective studies describing tuberculous meningitis (TBM) IRIS; only isolated cases [9–15] and 1 case series of neurological tuberculosis IRIS [8, 16] have been published. Although consensus now exists that ART should be started early (around 2 weeks) in HIV/tuberculosis-coinfected patients with severe immunosuppression, a potential exception is TBM because of the perceived risk of TBM-IRIS [17, 18]. However, the frequency and severity of this complication are not well documented and no means exist to predict the syndrome.

We therefore investigated clinical and laboratory findings in ART-naive HIV-infected patients who presented with TBM. We undertook serial cerebrospinal fluid (CSF) sampling in patients who did and did not develop TBM-IRIS.

## MATERIALS AND METHODS

### Setting

This prospective, observational study was performed at GF Jooste Hospital, a public sector referral hospital in Cape Town. The hospital serves a low-income, high-density population in which the tuberculosis notification rate exceeds 1.5% per year with 70% of tuberculosis cases coinfected with HIV [19].

### Participants

ART-naive HIV-infected patients aged ≥18 years presenting with meningitis from March 2009 through October 2010 were screened for study inclusion. HIV infection was diagnosed using 2 rapid HIV antibody tests and confirmed by HIV load. Definite TBM was diagnosed when acid-fast bacilli were seen, or when *M. tuberculosis* was cultured from CSF. Probable TBM was diagnosed when a patient showed clinical, laboratory, and radiological features of TBM in the absence of other infective causes for presentation [20]. Paradoxical TBM-IRIS was diagnosed according to a published definition for tuberculosis IRIS modified for meningitis [7, 8]. The definition had 3 components: (1) TBM diagnosis before starting ART and improvement on tuberculosis treatment prior to ART initiation; (2) onset of TBM-IRIS manifestations (ie, new, recurrent, or worsening clinical features of TBM) within 3 months of ART initiation; and (3) exclusion of alternative causes for clinical deterioration.

Patients were ineligible if they had a contraindication to lumbar puncture, including unequal pressures between individual brain compartments on brain imaging, or severe TBM (ie, modified British Medical Research Council [BMRC] grade III disease severity) [21]. The University of Cape Town Research Ethics Committee approved the study and written informed consent was obtained from all patients or their relatives.

### Procedures

Demographic data and history of tuberculosis disease, HIV infection, and other systemic illnesses and medications were recorded. Patients underwent general physical and neurological examination. Chest radiography, phlebotomy, and lumbar puncture were performed. In patients with suspected raised intracranial pressure or focal neurological deficits, brain computed tomography scanning was performed prior to lumbar puncture. CSF analysis included biochemistry, cytology, microbiology (including microscopy and culture for fungi and pyogenic bacteria), syphilis serology, HIV load, and *Cryptococcus* latex agglutination titer. Ziehl-Neelsen staining of sediment and *M. tuberculosis* culture was performed. If mycobacteria were cultured from CSF, tuberculosis polymerase chain reaction (PCR; Genotype MTBDRplus, Hain Lifesciences) was performed to determine susceptibility to rifampicin and isoniazid. CSF varicella zoster virus PCR was performed if the etiology was suspected. CSF was also stored at −80°C and analyzed for a range of inflammatory markers on the Bio-Plex platform (Bio-Rad Laboratories, Hercules, CA) using customized Milliplex kits (Millipore, St Charles, MO) according to the manufacturer's instructions.

At TBM diagnosis, patients started tuberculosis treatment according to national guidelines [22] and prednisone (1.5 mg/kg/day). After 2 weeks of treatment and prior to initiation of ART, patients were assessed for improvement on tuberculosis treatment. The initial ART regimen was stavudine, lamivudine, and efavirenz. Later during the study, tenofovir replaced stavudine according to revised national guidelines. CSF investigations performed at TBM diagnosis were repeated at the time of ART initiation, 2 weeks later, and at time of TBM-IRIS presentation and 2 weeks thereafter. Prednisone was reduced to 0.75 mg/kg/day 4 weeks after starting ART and discontinued 2 weeks thereafter, unless the patient developed TBM-IRIS. At TBM-IRIS presentation, investigations were performed to exclude alternative causes of deterioration. Prednisone was either recommenced or the dose increased. Patients were followed for the duration of tuberculosis treatment (9 months); routine visits were at 2 weeks, 4 weeks, 6 weeks, 12 weeks, 6 months, and 9 months after TBM diagnosis. Patients were seen more frequently during deterioration.

### Statistical Analysis

Statistical analysis was performed using the GraphPad Prism version 5, R version 2.14.1, and StatXact version 9 software packages. Categorical variables were compared using χ^2^ or Fisher exact test. Continuous variables were compared between groups and time points within groups, using the Wilcoxon rank sum and Wilcoxon matched pairs tests, respectively. Adjusted relative risks (RRs) were evaluated using Cochran-Mantel-Haenszel tests and tests of homogeneity when considering categorical risk factors. Log-binomial models were fitted to continuous risk factors. Significance testing was done using 2-sided *P* values with *P* < .05 taken as significant.

The predictive accuracy of CSF neutrophil counts at TBM diagnosis for TBM-IRIS was assessed using nonparametric area under the receiver operating characteristic curve (AUC). Additionally, a model to predict TBM-IRIS risk was developed from 5 prespecified cytokines measured in CSF at time of TBM diagnosis. Interleukin 6 (IL-6), interleukin 10, interleukin 12p40, interferon gamma (IFN-γ), and tumor necrosis factor alpha (TNF-α) were selected as candidate markers of TBM-IRIS based on previous studies [23, 24]. We prespecified the analysis for evaluating the multivariate cytokine model as follows. Significant cytokines comparing TBM-IRIS and non-TBM-IRIS using Wilcoxon rank sum tests were selected for a logistic regression model. Nonsignificant cytokines were dropped, resulting in a final model. The entire model building process was evaluated using leave-one-out cross-validation, the bootstrap method [25], and the permutation test to provide a cross-validated (nonparametric) estimate of the AUC, *P* values, and 95% confidence intervals (CIs). As a secondary analysis, we examined whether the addition of CSF neutrophils and/or lymphocyte counts would improve the model's predictive ability.

## RESULTS

### TBM Presentation

**Figure 1.**
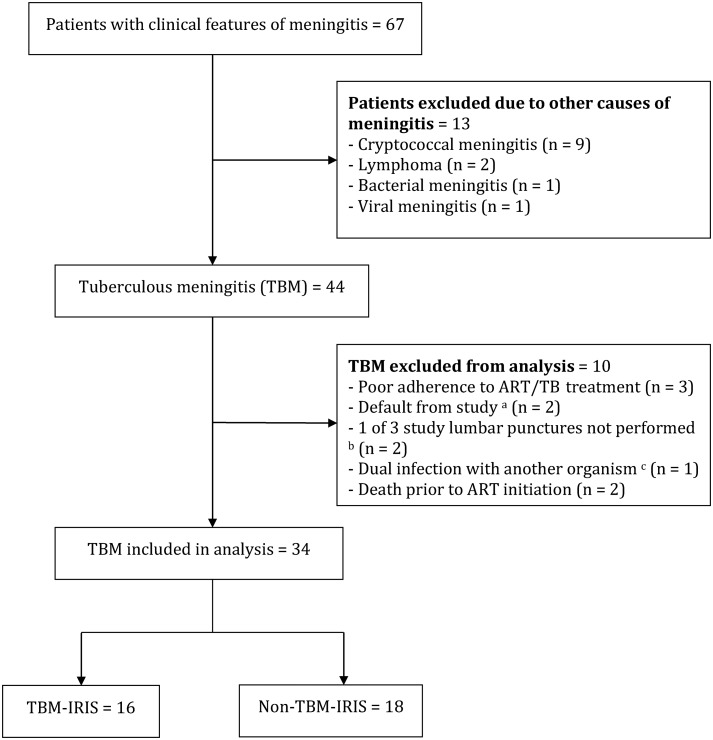
Flow diagram of patients with features of meningitis (eg, headache, confusion, vomiting, and/or neck stiffness) screened for study inclusion. ^a^Patients defaulted within 3 months of starting antiretroviral therapy (ART). ^b^Time points of lumbar punctures include tuberculous meningitis (TBM) diagnosis, ART initiation, and 2 weeks after starting ART. ^c^Cerebrospinal fluid varicella-zoster virus polymerase chain reaction was performed in 1 patient with who had shingles at time of TBM presentation, which was positive. Abbreviations: ART; antiretroviral therapy, IRIS; immune reconstitution inflammatory syndrome; TB; tuberculosis; TBM, tuberculous meningitis.

The final diagnoses and reasons for exclusion of patients with meningitis are shown in Figure 1. Thirty-four patients were included in the final analysis; 15 (44%) were female and the median age was 33 years (interquartile range [IQR], 29–44). Sixteen patients (47%) developed TBM-IRIS (TBM-IRIS patients) whereas 18 did not (non-TBM-IRIS patients). Tables 1 and 2 show the demographic and baseline characteristics of these 2 groups. Baseline characteristics were similar between groups, although patients who developed TBM-IRIS had a longer duration of symptoms (median, 19 vs 9 days) and more frequent features of extrameningeal tuberculosis, such as chest symptoms (81% vs 44%) and chest radiographic abnormalities (81% vs 50%). Five TBM-IRIS patients (31%) and 3 non-TBM-IRIS patients (17%) developed features of extrameningeal tuberculosis IRIS.

**Table 1.** Demographic and Baseline Characteristics of Patients Who Developed Tuberculous Meningitis Immune Reconstitution Inflammatory Syndrome and Those Who Did Not

	TBM-IRIS	Non-TBM-IRIS
	No. (%)	No. (%)
Female	9 (56)	6 (33)
Age, y, median (IQR)	33 (30–46)	31 (25–41)
Duration between tuberculosis treatment and ART, d, median (IQR)^a^	15 (14–16)	15 (14–20)
Previous tuberculosis	5 (31)	6 (33)
Neurological symptoms		
Duration of neurological symptoms, d, median (IQR)	19 (6–31)	9 (6–21)
Nausea/vomiting	13 (81)	8 (44)
Headache	16 (100)	14 (78)
Visual disturbances	6 (38)	5 (28)
Confusion	7 (44)	6 (33)
Neck pain/stiffness	13 (81)	14 (78)
Systemic symptoms		
Chest symptoms	13 (81)	8 (44)
Night sweats	12 (75)	8 (44)
Abdominal symptoms	9 (56)	5 (28)
Weight loss	14 (88)	12 (67)
Clinical findings		
Body mass index, median (IQR)	18.4 (17.2–23.3)	20.7 (19.0–23.3)
BMRC disease grade 1^b^	7 (44)	11 (61)
Focal neurological signs^c^	4 (25)	3 (17)
Blood investigations		
Sodium, mmol/L, median (IQR)	123 (121–129)	130 (128–134)
Hemoglobin, g/dL, median (IQR)	10.2 (8.5–11.7)	12.4 (9.3–13.4)
Other investigations		
CXR abnormalities	13 (81)	9 (50)
Features of extra CNS tuberculosis^d^	14 (88)	12 (67)
Abdominal ultrasound, abnormal/number performed	5/5 (100)	6/8 (75)
Brain CT abnormal/number performed^e^	4/5 (80)	5/9 (56)

Data are presented as No. (%) unless otherwise specified. Definitions for TBM-IRIS and non-TBM-IRIS are taken from [16] and [18], respectively.

Abbreviations: ART, antiretroviral therapy; BMRC, British Medical Research Council; CNS, central nervous system; CT, computed tomography; CXR, chest radiograph; IQR, interquartile range; TBM-IRIS, tuberculous meningitis immune reconstitution inflammatory syndrome.

^a^ ART regimens included stavudine (D4T), lamivudine (3TC), and efavirenz (EFV; n = 19); tenofovir, 3TC, and EFV (n = 9); zidovudine, 3TC, and EFV (n = 5); and D4T, 3TC, and lopinavir/ritonavir (n = 1).

^b^ BMRC grade I: Glasgow Coma Scale (GCS) score of 15 with no focal neurologic signs; grade II: GCS score of 11–14 or GCS of 15 with focal neurologic signs; grade III: GCS score of ≤10 [21].

^c^ Including cranial nerve palsies (n = 4), hemiparesis (n = 1), cerebellar signs (n = 2).

^d^ Including number of patients with 1 or more of the following: chest radiograph or abdominal ultrasound features of tuberculosis and acid-fast bacilli seen or *M. tuberculosis* cultured from specimen other than cerebrospinal fluid.

^e^ Including features compatible with TBM: hydrocephalus, meningeal enhancement, tuberculoma, and infarct.

**Table 2.** Serial **Blood and Cerebrospinal Fluid Findings in Patients Who Developed Tuberculous Meningitis Immune Reconstitution Inflammatory Syndrome (TBM-IRIS) and Those Who Did Not (non-TBM-IRIS)**

	TBM-IRIS	Non-TBM-IRIS	
	Median (IQR)	Median (IQR)	*P* Value^c^
Blood			
C-reactive protein, mg/L			
TBM diagnosis	45 (13–98)	25 (1–71)	.22
ART start	6 (4–15)	8 (1–20)	.64
2 wk after ART start	56 (20–105)	14 (6–63)	.07
CD4 count, cells/μL			
TBM diagnosis	131 (52–169)	102 (69–278)	.79
ART start	93 (65–158)	145 (64–231)	.40
2 wk after ART start	158 (139–193)	178 (103–261)	.68
HIV load, log_10_			
TBM diagnosis	5.39 (4.75–6.16)	5.60 (4.76–5.72)	.83
ART start	5.61 (5.26–6.26)	5.30 (4.90–6.04)	.13
2 wk after ART start	3.15 (2.50–3.36)	2.71 (2.43–2.98)	.13
Cerebrospinal fluid			
Protein, g/L			
TBM diagnosis	2.70 (1.80–5.11)	1.88 (1.28–3.31)	.24
ART start	1.63 (1.22–2.67)	0.94 (0.82–1.63)	.03
2 wk after ART start/IRIS presentation^a^	3.11 (1.99–22.83)	0.61 (0.37–1.76)	<.001
2 wk after IRIS presentation^b^	2.62 (2.24–12.74)	…	…
Glucose (CSF blood ratio)			
TBM diagnosis	0.24 (0.15–0.31)	0.47 (0.28–0.57)	.01
ART start	0.51 (0.43–0.55)	0.48 (0.38–0.57)	.63
2 wk after ART start/IRIS presentation^a^	0.39 (0.33–0.43)	0.53 (0.37–0.59)	.05
2 wk after IRIS presentation^b^	0.38 (0.34–0.48)	…	…
Neutrophil counts, ×10^6^/L			
TBM diagnosis	50 (15–86)	3 (0–44)	.02
ART start	1 (0–4)	0 (0–5)	.32
2 wk after ART start/IRIS presentation^a^	42 (17–244)	0 (0–3)	<.001
2 wk after IRIS presentation^b^	3 (0–12)	…	…
Neutrophil proportion, % of cells per sample			
TBM diagnosis	36 (6–53)	0 (0–12)	.009
ART start	1 (1–11)	0 (0–18)	.61
2 wk after ART start/IRIS presentation^a^	9 (4–22)	1 (0–33)	.35
Lymphocyte counts, ×10^6^/L			
TBM diagnosis	218 (63–366)	93 (24–274)	.36
ART start	110 (44–225)	41 (18–79)	.02
2 wk after ART start/IRIS presentation^a^	177 (69–363)	25 (6–52)	<.001
2 wk after IRIS presentation^b^	147 (98–405)	…	…
HIV load, log_10_			
TBM diagnosis	6.35 (5.80–6.73)	5.60 (4.81–6.54)	.05
ART start	5.56 (5.16–5.95)	5.40 (4.93–5.82)	.27
2 wk after ART start	3.00 (2.24–3.67)	2.55 (2.42–2.80)	.21

Definitions for TBM-IRIS and non-TBM-IRIS are taken from [16] and [18], respectively.

Abbreviations: ART, antiretroviral therapy; CSF, cerebrospinal fluid; HIV, human immunodeficiency virus; IQR, interquartile range; IRIS, immune reconstitution inflammatory syndrome; TBM, tuberculous meningitis.

^a^ For TBM-IRIS patients, results are reported for lumbar puncture performed at TBM-IRIS presentation, which occurred a median of 14 days after ART initiation.

^b^ In 5 TBM-IRIS patients, lumbar puncture was not performed 2 weeks after TBM-IRIS because of death (n = 1), contraindication to lumbar puncture (n = 3), and patient admitted to alternative facility (n = 1).

^c^ *P* < .05 was considered statistically significant.

### TBM-IRIS Presentation

Features of TBM-IRIS developed a median of 14 days (IQR, 4–20) after starting ART. Symptoms and signs included new or worsening headache (n = 12), confusion (n = 6), neck pain/stiffness (n = 11), generalized tonic-clonic seizures (n = 4), vomiting (n = 5), paraparesis (n = 3), myoclonic jerks (n = 1), dysconjugate eye movements (n = 1), and aphasia (n = 1). At time of TBM-IRIS presentation, 15 patients underwent brain imaging, including computed tomography (n = 14) or magnetic resonance imaging (n = 1). Imaging showed features of TBM in 14 of these patients. Magnetic resonance imaging of the spine was performed in 2 patients with paraparesis; both had features of radiculomyelitis (Supplementary Figure 1).

### Serial Blood and CSF Findings

**Figure 2.**
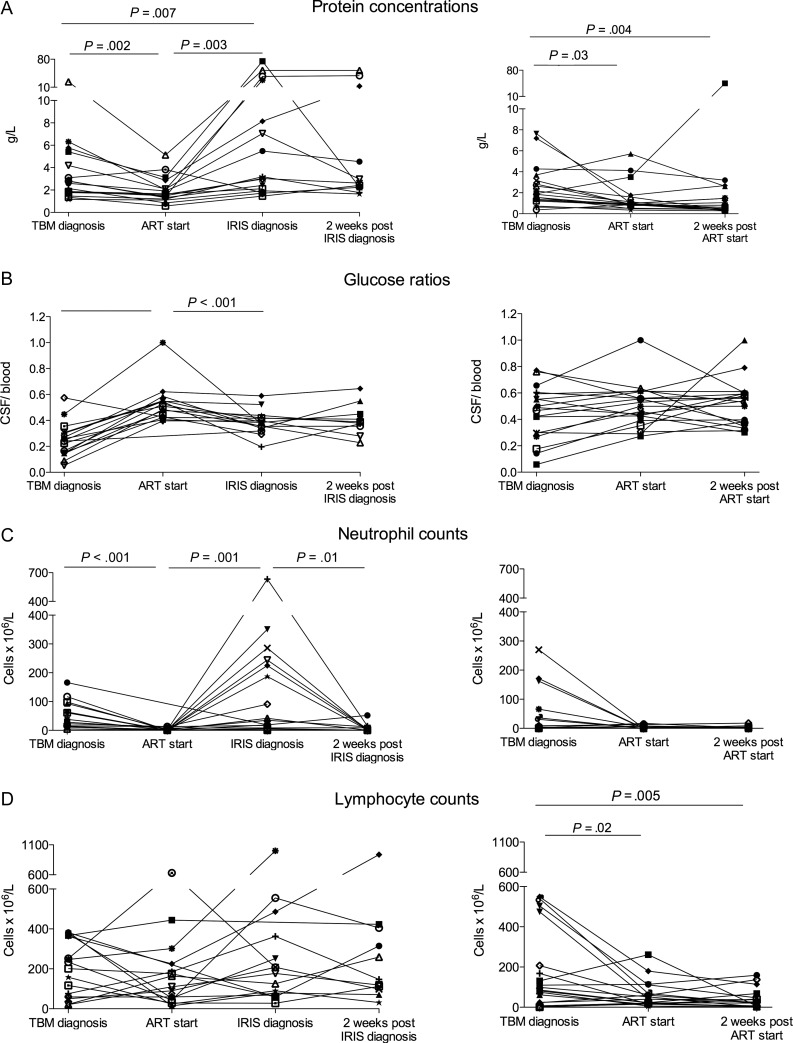
Serial cerebrospinal fluid (CSF) findings in patients who developed tuberculous meningitis immune reconstitution inflammatory syndrome (left), and those who did not (right), including protein concentrations (*A*), CSF to blood glucose ratios (*B*), neutrophil counts (*C*), and lymphocyte counts (*D*). Significant differences (*P* < .05) between time points within groups are indicated. Abbreviations: ART; antiretroviral therapy, CSF cerebrospinal fluid; IRIS; immune reconstitution inflammatory syndrome; TBM, tuberculous meningitis.

Baseline blood investigations were similar between TBM-IRIS and non-TBM-IRIS patients with the exception of serum sodium concentrations, which were lower in TBM-IRIS patients (median, 123 vs 130 mmol/L, *P* = .01, Table 1). Table 2 and Figure 2 show serial blood and CSF findings. There was a significant rise in CD4 counts between starting ART and 2 weeks later in both TBM-IRIS (median, 93–158 cells/μL, *P* = .009) and non-TBM-IRIS (median, 145–178 cells/μL, *P* = .04) patients. Between these time points, blood and CSF HIV loads decreased significantly (*P* < .001) in both groups.

At TBM diagnosis, TBM-IRIS patients had higher CSF cell counts, in particular neutrophils (median, 50 vs 3 cells ×10^6^/L, *P* = .02, Table 2). Similarly, neutrophil percentages from individual samples were higher in TBM-IRIS patients compared with non-TBM-IRIS patients (median, 36% vs 0%, *P* = .009). CSF to blood glucose ratios were lower in TBM-IRIS patients (median, 0.24 vs 0.47, *P* = .005). In both groups, CSF parameters initially improved on tuberculosis treatment (Table 2 and Figure 2). However, at TBM-IRIS presentation, TBM-IRIS patients showed findings of recurrent inflammation. In this group, lymphocyte and neutrophil counts at TBM-IRIS presentation were similar, and protein concentrations higher, compared with the same parameters at TBM diagnosis (median protein, 3.11 g/L at TBM-IRIS vs 2.70 g/L at TBM diagnosis, *P* = .007).

*Mycobacterium tuberculosis* was cultured from the CSF of 15 TBM-IRIS patients (94%) and 6 non-TBM-IRIS patients (33%) at TBM diagnosis; the risk of developing TBM-IRIS if CSF *M. tuberculosis* culture was positive at this time point was 71.4% (15/21), compared with a risk of 7.7% (1/13), corresponding to an RR of 9.3 (95% CI, 1.4–62.2, *P* = .004). Additional analyses considered the RRs of culture positivity adjusting for the following known or potential risk factors for tuberculosis IRIS: baseline viral load (median, ≥330 000 copies/mL, equivalent to log_10_ = 5.52, vs <330 000), CD4 count (median, ≤137 cells/μL vs >137), evidence of disseminated disease in the form of an abnormal chest radiograph suggesting miliary disease, and duration of illness (median duration, ≥2 weeks vs <2 weeks). *Mycobacterium tuberculosis* culture positivity remained a significant risk factor after adjusting for these factors (Supplementary Table 1). There was no evidence of differences in the RRs according to these factors after considering culture status. Some TBM-IRIS patients remained culture positive after 2 weeks (n = 7) and 4 weeks (n = 2) of tuberculosis treatment. No non-TBM-IRIS patients were culture positive after starting tuberculosis treatment. All cultures were fully drug sensitive with the exception of one, which was monoresistant to isoniazid.

### Analysis of Baseline CSF Neutrophil Count and Cytokine Concentrations to Predict TBM-IRIS

**Figure 3.**
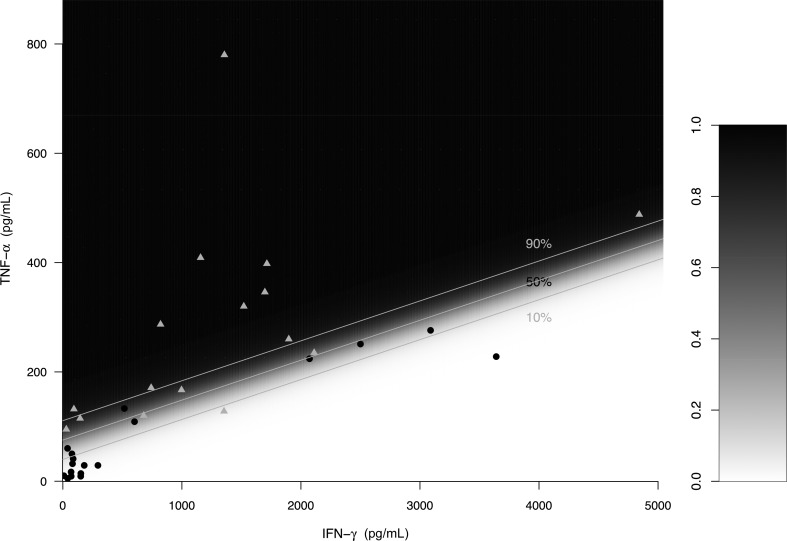
Predictive model for tuberculous meningitis immune reconstitution inflammatory syndrome (TBM-IRIS; patients depicted by gray triangles) and non-TBM-IRIS (patients depicted by black circles). Tumor necrosis factor–α and interferon-γ concentrations (pg/mL) are reported. Darker gray indicates greater probability of TBM-IRIS, while lighter gray indicates greater probability of not developing TBM-IRIS. Probabilities associated with shading are indicated by the legend. The middle line indicates 50% chance of TBM-IRIS, while the upper and lower gray lines indicate probabilities of 90% and 10%, respectively. Observe that when using the median line to classify patients as TBM-IRIS or non-TBM-IRIS, all but 2 TBM-IRIS and 1 non-TBM-IRIS patients are correctly classified. Several points in the lower left were moved marginally to the right so that all subjects are clearly identifiable. Abbreviations: IFN, interferon; TNF, tumor necrosis factor.

Concentrations of the prespecified cytokines from which the model to predict TBM-IRIS was developed are shown in Supplementary Table 2 and Supplementary Figure 2. The final multivariate logistic regression model included IFN-γ and TNF-α and produced a cross-validated AUC of 0.91 (95% CI, .53–.99, *P* = .02), indicating high diagnostic accuracy when jointly considering these 2 cytokines to differentiate TBM-IRIS from non-TBM-IRIS at time of TBM diagnosis. The odds ratio (OR) for TNF-α was 1.85 (per 10 pg/mL, *P* = .006), indicating an 85% increase in the odds of IRIS for every 10 pg/mL increase in TNF-α (after adjusting for IFN-γ). The OR for IFN-γ was 0.64 (per 100 pg/mL, *P* = .01), indicating decreased odds of IRIS with increasing IFN-γ (after adjusting for TNF-α). Figure 3 provides a heatmap representation of the predicted probabilities of the resulting model with the observed values overlaid. Neutrophil counts produced an AUC of 0.72 (95% CI, .54–.90, *P* = .03), indicating modest discriminatory accuracy for TBM-IRIS. However, including CSF neutrophil and lymphocyte counts in the model did not improve its ability to predict TBM-IRIS.

### Management and Outcome

In 13 patients prescribed prednisone (0.75–1.5 mg/kg/day), the dose was increased at TBM-IRIS diagnosis. Prednisone was restarted at a dose of 1.5 mg/kg/day in the other 3 TBM-IRIS patients. The median total duration of corticosteroid treatment in TBM-IRIS patients was 109 days (IQR, 69–141) compared with 35 days (IQR, 20–43) in non-TBM-IRIS patients. ART was interrupted during TBM-IRIS in 1 patient because of brainstem involvement. This patient made a full recovery and had no recurrent symptoms after recommencement of ART under prednisone cover. At 9 months’ follow-up, all non-TBM-IRIS patients were alive (including 1 who had defaulted study follow-up but continued tuberculosis treatment from a primary care tuberculosis clinic), 2 had marked cognitive impairment (international HIV dementia scale <10) [26], and 1 patient had marked cognitive impairment with residual hemiparesis. Twelve IRIS patients (75%) were alive at 9 months’ follow-up, 2 patients showed marked cognitive impairment, 1 patient defaulted study follow-up but was alive, and 1 had marked cognitive impairment, residual hemiparesis, and hearing impairment. Death occurred in 4 (25%) TBM-IRIS patients at 33, 53, 60, and 118 days after TBM diagnosis and was related to TBM-IRIS in 2 patients. The 2 other deaths were due to a road traffic accident (n = 1) and unknown cause (n = 1). Kaplan-Meier survival analysis by TBM-IRIS vs non-IRIS (with a log-rank hypothesis test of the difference in survival between these 2) was nonsignificant.

## DISCUSSION

This is the first prospective study of TBM-IRIS. In our cohort, tuberculosis IRIS presenting as TBM-IRIS (47%), as well as tuberculosis IRIS involving any organ system (56%), was more frequent than in previous studies [2–4, 27]. Extrapulmonary tuberculosis is a risk factor for tuberculosis IRIS [14, 28], and our cohort included only patients with TBM. A shorter interval between starting tuberculosis treatment and ART (which was 2 weeks in our study) similarly increases the risk of tuberculosis IRIS [2–4]. The high TBM-IRIS incidence we observed is striking considering that all TBM-IRIS patients were taking prednisone (1.5 mg/kg/day) at time of ART initiation and 13 of these patients (81%) were taking prednisone (0.75–1.5 mg/kg/day) at the time of developing TBM-IRIS. Adjunctive corticosteroids have been shown to reduce mortality in TBM and tuberculosis pericarditis, presumably by reducing pathological host immune responses [21, 29]. In paradoxical tuberculosis IRIS, the symptomatic benefit of corticosteroids was demonstrated in a randomized trial in which prednisone was compared to placebo [30]. For these reasons, we anticipated that prednisone would decrease the risk of tuberculosis IRIS.

TBM-IRIS was associated with a poor outcome; 2 patients (13%) died as a result of TBM-IRIS, all-cause mortality at 9 months was 25%, and 3 of 11 (27%) survivors examined at 9 months’ follow-up were severely disabled, compared with no deaths and 3 of 17 (18%) patients with severe morbidity in the non-TBM-IRIS group. Our findings are similar to a previous study performed in neurological tuberculosis IRIS [8]. The poor outcome in at least 44% of TBM-IRIS cases emphasizes the need to predict and prevent, and improve the treatment of, TBM-IRIS.

Low serum sodium concentration is associated with death in HIV-associated TBM [31]. In our study, serum sodium concentration was lower in TBM-IRIS patients compared with non-TBM-IRIS patients at the time of TBM diagnosis. This may reflect the higher degree of tuberculosis dissemination observed in TBM-IRIS patients, which could have contributed to their risk of developing TBM-IRIS.

Our finding of an association between higher CSF neutrophils at TBM presentation and subsequent development of TBM-IRIS provides important and novel insight into the pathogenesis of tuberculosis IRIS. Not only were neutrophil counts higher in TBM-IRIS patients compared with non-TBM-IRIS patients, but neutrophil percentages for individual patients were similarly raised in TBM-IRIS. The neutrophil counts showed dynamic fluctuations over time in TBM-IRIS patients with a marked decrease on tuberculosis treatment, and a striking increase at TBM-IRIS onset. Similar changes in lymphocyte counts were not observed. Studies of tuberculosis IRIS have hitherto focused on the contribution of helper T-cell type 1 lymphocyte responses [32, 33]. However, a role for myeloid cells in tuberculosis IRIS is suggested by a case report of a patient who died from unmasking pulmonary tuberculosis IRIS; postmortem histological examination of diseased lung showed a marked macrophage infiltrate [34]. We have found cytokines of predominantly myeloid origin (IL-6 and TNF-α) to be consistently elevated in patients with tuberculosis IRIS, compared with those who did not develop IRIS [23]. Oliver et al [6] also reported an association between plasma cytokines (interleukin 18) and chemokines (CXCL-10) of the innate immune system and tuberculosis IRIS. In an animal model, immune reconstitution following transfer of mycobacteria-specific CD4 T cells to T-cell–deficient mice infected with *Mycobacterium avium* was associated with marked increases of both blood and lung CD11b cells (likely representing inflammatory monocytes and neutrophils) [35]. Our results suggest that neutrophils contribute to tuberculosis IRIS pathogenesis. The combination of high CSF TNF-α and low IFN-γ concentrations at the time of TBM diagnosis predicted TBM-IRIS in this cohort. Simmons et al [36] reported a negative correlation between CSF IFN-γ and mortality in HIV-infected patients with TBM. Conversely, a positive correlation was found between CSF IFN-γ and TNF-α concentrations and TBM disease severity by others [37].

Several studies have shown an association between disseminated and extrapulmonary tuberculosis and subsequent tuberculosis IRIS [14, 28, 38, 39]. At TBM diagnosis, CSF *M. tuberculosis* culture positivity, which reflects mycobacterial antigen load, was a major risk factor for developing TBM-IRIS (RR = 9.3). Furthermore, 7 TBM-IRIS patients (44%) were persistently CSF *M. tuberculosis* culture positive after 2 weeks of tuberculosis treatment and 2 patients (13%) remained culture positive after 4 weeks of tuberculosis treatment. This strongly supports the inference that a high *M. tuberculosis* bacillary load at time of starting ART is a risk factor for tuberculosis IRIS [40]. The findings suggest it important to optimize tuberculosis treatment prior to starting ART in patients at high risk of developing TBM-IRIS.

We acknowledge several limitations. Because of the relatively small sample size, the study may not have been powered to detect further differences between IRIS and non-TBM-IRIS patients. Only patients with less severe disease (BMRC TBM grade 1 and 2) and those without contraindications to lumbar puncture were enrolled, resulting in the exclusion of a significant proportion of TBM patients presenting in our setting [41]; our results may therefore not be generalizable to ART-naive patients presenting with severe HIV-associated TBM. The model to predict TBM-IRIS needs further validation and exploration with independent data.

In conclusion TBM-IRIS complicated the course of treatment of HIV-associated TBM in nearly half our patients, despite the use of adjunctive corticosteroid therapy. The manifestations were severe, fatal in 2 cases. The occurrence of TBM-IRIS associated with CSF *M. tuberculosis* culture positivity and a high neutrophil count at both baseline and at the time of TBM-IRIS. The baseline relationship between CSF TNF-α and IFN-γ predicted TBM-IRIS. These observations provide novel insight into the pathogenesis of this condition and provide rationale to individualize ART beyond 2 weeks in this devastating, partly iatrogenic, condition.

## Supplementary Data

Supplementary materials are available at *Clinical Infectious Diseases* online (http://www.oxfordjournals.org/our_journals/cid/). Supplementary materials consist of data provided by the author that are published to benefit the reader. The posted materials are not copyedited. The contents of all supplementary data are the sole responsibility of the authors. Questions or messages regarding errors should be addressed to the author.

Supplementary Data
