# Supplementary material for: Frequency, Severity, and Prediction of Tuberculous Meningitis Immune Reconstitution Inflammatory Syndrome
Source: Clin Infect Dis. 2012 Oct 24;56(3):450–60. doi: 10.1093/cid/cis899 (PMC3540040; doi:10.1093/cid/cis899)
Supplement: Supplementary Data [file supp_56_3_450__index.html]

Frequency, severity and prediction of tuberculous meningitis immune reconstitution inflammatory syndrome — Frequency, Severity, and Prediction of Tuberculous Meningitis Immune Reconstitution Inflammatory Syndrome — Frequency, Severity, and Prediction of Tuberculous Meningitis Immune Reconstitution Inflammatory Syndrome — Supplementary Data 

# Frequency, Severity, and Prediction of Tuberculous Meningitis Immune Reconstitution Inflammatory Syndrome

## Supplementary Data

Supplementary Data

**Files in this Data Supplement:**

- Supplementary Figure 1 - doc file
- Supplementary Figure 2 - doc file
- Supplementary Table 1 - doc file
- Supplementary Table 2 - doc file
