# Supplementary material for: Frequency, Severity, and Prediction of Tuberculous Meningitis Immune Reconstitution Inflammatory Syndrome
Source: Clin Infect Dis. 2012 Oct 24;56(3):450–60. doi: 10.1093/cid/cis899 (PMC3540040; doi:10.1093/cid/cis899)
Supplement: Supplementary Data [file supp_cis899_cis899supp_fig1.doc]

**Supplementary Figure 1. Brain and spine imaging of patients with TBM-IRIS**

A, B, and C, Post contrast brain computed tomography (CT) shows extensive focal leptomeningeal enhancement with surrounding edema in 3 patients at time of TBM-IRIS presentation. D, Sagittal T2-weighted magnetic resonance image of lumbar spine shows thickened nerve roots and abnormal conus medullaris signal consistent with radiculomyelitis in a patient with paraparesis at time of TBM-IRIS. E and F, Post contrast CT brain images from one patient shows marked increase in basal meningeal enhancement at time of TBM-IRIS presentation (F), compared to time of TBM presentation (E).
