# Supplementary material for: Frequency, Severity, and Prediction of Tuberculous Meningitis Immune Reconstitution Inflammatory Syndrome
Source: Clin Infect Dis. 2012 Oct 24;56(3):450–60. doi: 10.1093/cid/cis899 (PMC3540040; doi:10.1093/cid/cis899)
Supplement: Supplementary Data [file supp_cis899_cis899supp_fig2.doc]

**Supplementary Figure 2. Cerebrospinal fluid concentrations of cytokines included in a model to predict tuberculous meningitis immune reconstitution inflammatory syndrome (TBM-IRIS).**

Results are reported for lumbar puncture performed at TBM diagnosis in patients who developed TBM-IRIS and those who did not (non-TBM-IRIS). Medians +/- IQR are indicated.
