# Supplementary material for: Frequency, Severity, and Prediction of Tuberculous Meningitis Immune Reconstitution Inflammatory Syndrome
Source: Clin Infect Dis. 2012 Oct 24;56(3):450–60. doi: 10.1093/cid/cis899 (PMC3540040; doi:10.1093/cid/cis899)
Supplement: Supplementary Data [file supp_cis899_cis899supp_table1.doc]

**Supplementary Table 1: Risks of TBM-IRIS for cerebrospinal fluid *M. tuberculosis* culture positive relative to culture negative patients adjusting for other risk factors**

| **Risk Factor** | **Adjusted relative risk** | **p-value** |
| --- | --- | --- |
| Baseline blood HIV viral load ≥ 330,000 copies/ml  (equivalent to log_10_=5.52) | 10.6 | 0.002 |
| Baseline CD4 ≤ 137 cells/μL | 8.0 | <0.001 |
| Abnormal chest radiograph at TBM presentation | 8.7 | <0.001 |
| Neurological symptom duration (≥ 2 weeks) | 7.8 | <0.001 |
