# Supplementary material for: Frequency, Severity, and Prediction of Tuberculous Meningitis Immune Reconstitution Inflammatory Syndrome
Source: Clin Infect Dis. 2012 Oct 24;56(3):450–60. doi: 10.1093/cid/cis899 (PMC3540040; doi:10.1093/cid/cis899)
Supplement: Supplementary Data [file supp_cis899_cis899supp_table2.doc]

**Supplementary Table 2: Cerebrospinal fluid cytokine concentrations included in a model to predict tuberculous meningitis immune reconstitution inflammatory syndrome (TBM-IRIS).**

|  | **TBM-IRIS (16)** | | **Non-TBM-IRIS (18)** | |  |
| --- | --- | --- | --- | --- | --- |
| Cytokine (pg/ml) | Median | (IQR) | Median | (IQR) | P-value |
| Tumor necrosis factor-α | 248 | (129-385) | 37 | (13-156) | <0.001 |
| Interferon-γ | 1256 | (695-1710) | 18 | (4-970) | 0.007 |
| Interleukin-10 | 120 | (82-189) | 60 | (20-214) | 0.11 |
| Interleukin-6 | 8817 | (1668-10554) | 71 | (16-3939) | <0.001 |
| Interleukin-12p40 | 43 | (12-108) | 4 | (0-57) | 0.09 |

**Key:**

IQR; interquartile range

Results are reported for patients who developed TBM-IRIS and those who did not (non-TBM-IRIS); Results are reported for lumbar puncture performed at time of TBM diagnosis. A p-value of < 0.05 was considered to be statistically significant.
